# Case Report: Clinical features and management of anti-mGluR1 encephalitis: case illustration and review of the literature

**DOI:** 10.3389/fnint.2025.1580767

**Published:** 2025-07-23

**Authors:** Min Deng, Jing Xiong, Zhaohong Kong, Xufeng Wang, Tao Li

**Affiliations:** ^1^Renmin Hospital of Wuhan University, Wuhan, China; ^2^Hubei Provincial Hospital of Traditional Chinese Medicine, Wuhan, Hubei, China

**Keywords:** mGluR1, encephalitis, treatment, clinical features, ofatumumab

## Abstract

**Background:**

Anti-metabotropic glutamate receptor 1 (mGluR1) encephalitis is a rare autoimmune disease affecting cerebellar Purkinje cells. Only thirty-nine cases have been reported globally, with inconsistent documentation of treatments and outcomes. A systematic review is needed to identify prognostic factors and expand clinical understanding and treatment options.

**Methods:**

Observational follow-up data of anti-mGluR1 encephalitis cases were collected. All anti-mGluR1 encephalitis cases published in the PubMed and Google Scholar databases in English before November 1, 2024 were included. Clinical information and possible predictive factors from both current and previously reported cases were statistically analyzed.

**Results:**

We present a case of anti-mGluR1 encephalitis successfully treated with ofatumumab. During the patient’s initial episode, she partially recovered after first-line treatment. She experienced a relapse 6 months later and was treated with ofatumumab, resulting in complete recovery. Forty cases of anti-mGluR1 encephalitis, including our case, were summarized. The prevalence was similar between men and women, with 50% of patients aged 40–59 years. The most common clinical manifestations were ataxia and dysarthria. Cerebrospinal fluid analysis showed normal white blood cell count and IgG index in 37.1% of patients. Almost half of the patients (48.6%) exhibited cerebellar atrophy on cerebral MRI scans at initial presentation or during follow-up. Only 25% of patients recovered completely. According to the modified Rankin Scale (mRS) scores at the last follow-up, patients with poor outcome (*n* = 13, 32.5%) had a lower proportion of first-line immunotherapy (62%, *P* = 0.017) and a longer follow-up time (median 36 months, *P* = 0.038).

**Conclusion:**

The peak incidence of anti-mGluR1 encephalitis occurs between ages of 40–59 years. More than one-third of patients have normal cell counts and IgG index in the cerebrospinal fluid. Therefore, patients suspected of having this encephalitis should be tested for the presence of anti-mGluR1 antibodies in serum and cerebrospinal fluid. Notably, the first-line immunotherapy may be a critical factor influencing clinical outcomes.

## 1 Introduction

Metabotropic glutamate receptors (mGluRs) are class C G-protein-coupled receptors involved in regulating neuronal excitability throughout the central nervous system. MGluR1 is abundant in the mammalian brain, with the rich expression observed in cerebellar Purkinje cells (Yamasaki et al., 2021). The cerebellum is essential for the control and coordination of movement, as well as certain cognitive functions. Purkinje cells are the output neurons in the cerebellar cortex and play a crucial role in various cerebellar processes (Yamasaki et al., 2021; Engbers et al., 2013). The mGluR1 is known to regulate both functional and structural plasticity as well as synaptic responses by triggering downstream signaling pathways (Hartmann et al., 2014).

Increasing evidence suggests that any interference or disruption in the mGluR1 signaling pathway can lead to cerebellar dysfunction in humans and animal models (Yamasaki et al., 2021; Kano and Watanabe, 2017). However, since the first report of cerebellar symptoms caused by mGluR1 antibodies in humans in 2000, only 39 cases of anti-mGluR1 encephalitis have been documented (Smitt et al., 2000; Marignier et al., 2010; Lancaster et al., 2011; Iorio et al., 2013; Lopez-Chiriboga et al., 2016; Yoshikura et al., 2018; Pedroso et al., 2018; Christ et al., 2019; Gollion et al., 2019; Chaumont et al., 2019; Spatola et al., 2020; Bien et al., 2020; Chandler et al., 2022; Wang et al., 2023; Vinke et al., 2022; Goh et al., 2022; Chen et al., 2024). Due to the scarcity of reported anti-mGluR1 encephalitis cases globally, comprehensive multicenter studies incorporating diverse ethnic populations with distinct genetic profiles are imperative to elucidate the full clinical spectrum, immunopathogenic mechanisms, and long-term prognostic outcomes of this autoimmune disorder. In this context, we present a case of anti-mGluR1 encephalitis in a patient who experienced a relapse within a short timeframe but achieved complete recovery following treatment with ofatumumab. Ofatumumab is a fully human anti-CD20 monoclonal antibody that was approved for marketing in 2009 for the treatment of chronic lymphocytic leukemia (Zhang, 2009). This represents the first reported case of anti-mGluR1 encephalitis successfully treated with ofatumumab, thereby expanding the known clinical phenotype of the disease. In this report, we summarized the clinical characteristics, therapeutic approaches, and clinical outcomes of patients with anti-mGluR1 encephalitis by reviewing previously reported cases and current data. Additionally, we analyze the factors influencing clinical outcomes, providing valuable insights into the management of this rare yet debilitating disorder.

## 2 Methods

### 2.1 Research design and data collection

Data from the current case and selected cases of anti-mGluR1 encephalitis were collected to analyze the clinical characteristics, tumor associations, and treatment responses associated with this disease. To identify factors influencing clinical outcomes, data including demographic information, prodromal symptoms, clinical manifestations, concomitant malignancies, severity at peak and last follow-up, diagnostic findings, treatment strategies, clinical outcomes and recurrence rates were analyzed in detail. In the current case, disease severity at peak and each follow-up visit were evaluated using the modified Rankin Scale (mRS). For these selected cases, mRS scores were utilized to assess severity at peak and last follow-up based on documented symptoms and clinical outcomes. Outcomes were classified into four categories: lack of improvement, partial recovery, complete recovery (return to baseline), or death. According to the mRS score at the last follow-up visit, cases were stratified into a good outcome group (mRS ≤ 2) and a poor outcome group (mRS > 2). Relapse was defined as the emergence of new neurological symptoms or the exacerbation of residual symptoms after at least 2 months of stability following initial treatment.

### 2.2 Literature review

We conducted a comprehensive search of PubMed and Google Scholar for all cases of autoimmune encephalitis associated with human anti-mGluR1 antibodies published before November 2024. The search terms used included [(mGluR1 OR metabotropic glutamate receptor 1 OR anti-mGluR1 OR anti-metabotropic glutamate receptor 1 OR mGluR1-antibody) and (encephalitis OR autoimmune encephalitis OR Ophelia syndrome)]. The study followed the preferred reporting items for systematic reviews and meta-analyses (PRISMA) guidelines.

All cases with anti-mGluR1 encephalitis published in English were included in the analysis. The diagnosis of anti-mGluR1 encephalitis was based on clinical findings and the detection of mGluR1 antibodies in either serum or cerebrospinal fluid (CSF). Cases were excluded according to the following criteria: (1) low titers of anti-mGluR1 antibodies in both serum and CSF (less than 1:10); (2) the presence of other coexisting antibodies that provided a more plausible explanation for the patient’s symptoms; and (3) studies involving non-human subjects exclusively. All authors independently assessed the eligibility of each article identified in the database search. Article inclusion was determined through a two-step process: initial screening of titles and abstracts, followed by a full-text review. All disagreements were resolved by consensus.

### 2.3 Ethical considerations and patient consents

This study was approved by the Ethics Committee of Renmin Hospital of Wuhan University. Informed consent for the use of this patient’s medical records and blood and CSF samples was obtained. All data in the study were strictly anonymous.

### 2.4 Statistical analyses

Statistical analyses and graphical representations were performed using SPSS version 25.0. Continuous variables were analyzed using the Mann-Whitney U test and presented as medians. Categorical variables were analyzed using Fisher’s exact test and expressed as proportions. In addition, we compared variables that may be associated with clinical outcomes between patients with poor prognosis and those with good prognosis. Differences between the two groups were estimated using odds ratios (OR) with corresponding 95% confidence intervals (CI). *P*-values were obtained by Z test, and *p* < 0.05 were considered statistically significant.

## 3 Results

### 3.1 Case illustration

**FIGURE 1:**
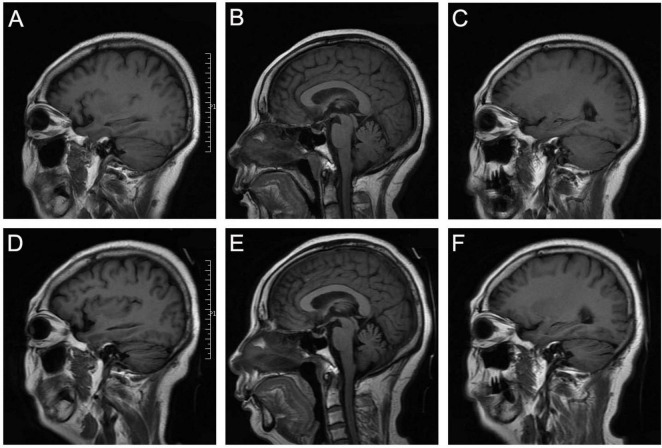
The radiology images. **(A-C)** Initial sagittal T1-weighted FLAIR sequences at baseline assessment. **(D-F)** Follow-up sagittal T1-weighted FLAIR sequences at terminal evaluation, demonstrating development of cerebellar atrophy.

A 58-year-old woman presented with dizziness and nausea following dental implant therapy in July 2023. One week later, she developed ataxia and became unable to stand without assistance, accompanied by dysarthria and visual disturbances. She denied any history of poisoning or family illness. Neurological examination revealed spontaneous nystagmus and positive bilateral finger to nose and heel knee tests. Blood cell count showed elevated leukocytes (13.05 × 10^9^/L), neutrophils (8.34 × 10^9^/L) and monocytes (1.23 × 10^9^/L). Additionally, she exhibited hypokalemia (2.87 mmol/L, reference range 3.6–5.2 mmol/L) and hypertriglyceridemia (3.49 mmol/L, reference value < 1.7 mmol/L). The whole blood mycobacterium tuberculosis specific T cell assay was positive. Vestibular testing demonstrated vertical downbeat nystagmus when gazing in the left, right, upward, and downward directions, along with positive Dix-Hallpike test and static positional test. Other laboratory findings, including antinuclear antibodies, liver function, renal function, blood glucose, hepatitis B virus, blood tumor markers, syphilis, and human immunodeficiency virus antibodies, were within normal limits. Brain magnetic resonance imaging (MRI) revealed cerebellar atrophy (Figure 1). MRI spectroscopy of the cerebellum showed no abnormalities. Electroencephalogram (EEG) and chest computed tomography (CT) scans were unremarkable.

**FIGURE 2:**
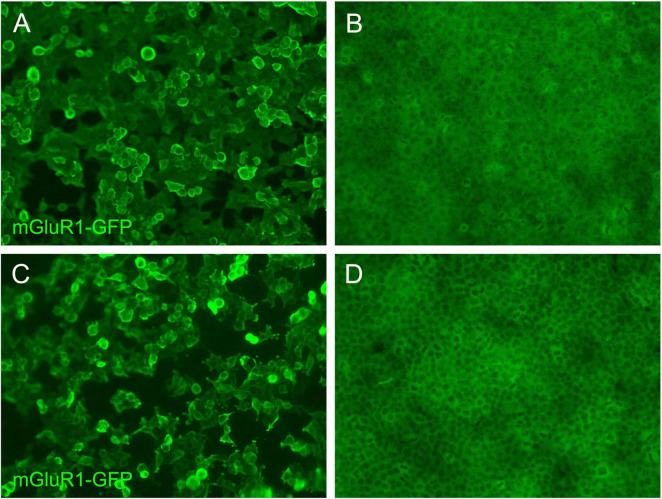
The fluorescence images of mGluR1 IgG positive and control in serum and cerebrospinal fluid. **(A)** Fluorescent antibody staining for expression of mGluR1 in the first blood of the patient. **(C)** Fluorescent antibody staining for expression of mGluR1 in the first CSF of the patient. Negative controls for serum **(B)** and CSF **(D)** respectively show background-level signals.

Analysis of CSF revealed normal intracranial pressure (120 mmH_2_O), elevated leukocyte count (120 × 10^6^/L, reference range (0–8) × 10^6^/L), increased protein level (0.49 g/L, reference range 0.15 mg/L–0.45 g/L) and normal glucose level (2.6 mmol/L, 2.5–4.4 mmol/L). The patient was diagnosed with autoimmune encephalitis and treated with intravenous immunoglobulin (IVIG, 0.4 mg/kg for 5 days). However, her ataxia symptoms worsened compared to baseline. The lumbar puncture was performed again. CSF analysis showed normal intracranial pressure (130mm H_2_O), further elevated leukocyte count (250 × 10^6^/L), increased protein level (0.42 g/L) and normal glucose level (2.74 mmol/L). The transfection cytology methods were used to detect autoimmune encephalitis antibodies (NMDAR, AMPAR1, AMPAR2, LGI1, CASPR2, GABABR, DPPX, IgLON5, GlyRα1, GABAARα1, GABAARβ3, mGluR1, mGluR5, D2R, Neurexin-3α, GAD65, KLHL11, gAChR, AQP4, MOG, GFAP) in CSF and serum. The mGluR1 IgG was detected in her serum (1:100,Figure 2A) and CSF (1:100,Figure 2C). Corresponding controls (Figures 2B, D) showed no immunostaining, confirming specificity. Following the treatment of intravenous methylprednisolone (IVMP, 1000 mg/d, reduced by half every 3 days for 15 days), she underwent lumbar puncture again. CSF analysis at this time showed normal intracranial pressure (120mm H_2_O), mildly elevated leukocytes (10 × 10^6^/L), normal protein level (0.22 g/L) and normal glucose level (2.7 mmol/L). Clinically, her symptoms of dizziness and ataxia partially recovered, and she was discharged. She was advised to continue oral IVMP (8 mg, QD) and mycophenolate (0.5 g, BID) treatment at home.

**FIGURE 3:**
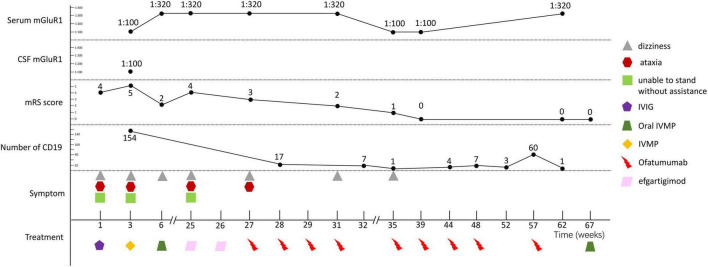
Schematic illustration of the disease course and follow-up.

The patient experienced a relapse in January 2024, presenting with dizziness and an inability to walk independently. The serum mGluR1 antibody was positive (1:320). She underwent two cycles of efgartigimod treatment over 15 days, resulting in only slight improvement in her symptoms. Subsequently, she received nine doses of ofatumumab. No drug allergies or adverse reactions were observed during the treatment. The symptoms of her dizziness and ataxia recovered completely. However, despite clinical recovery, her serum mGluR1 antibody titers remained positive during subsequent follow-up visits. At Week 67, an 8mg dose of oral IVMP was added to the treatment regimen. The clinical manifestations, diagnostic findings, treatment strategies and follow-up outcomes of the patients were detailed in Figure 3.

### 3.2 Review of the literature

**FIGURE 4:**
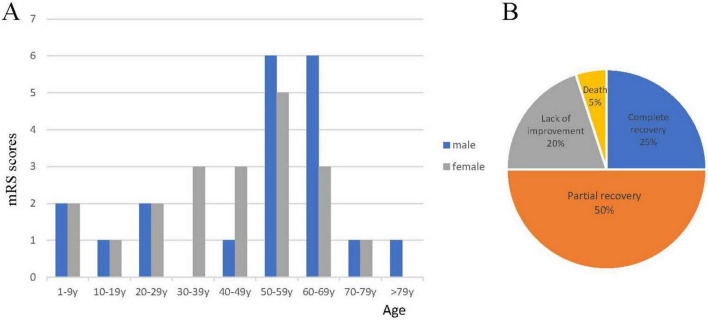
Demographics and clinical outcomes in all patients with anti-mGluR1 encephalitis. **(A)** The distribution of patients by age and sex in anti-mGluR1 encephalitis. **(B)** Proportion of outcomes in 40 patients.

We reviewed the clinical information of 39 previously reported cases and combined it with that of the patient in this study. The details were summarized in Table 1. The median age at onset of anti-mGluR1 encephalitis was 50.5 years (range 3–81 years), and females comprising 50% of cases. There was a peak in the prevalence of cases aged 50–69 years, accounting for 50% of all patients. The distribution of age and gender at onset was illustrated in Figure 4A. Prodromal symptoms were reported in 25% of patients, including headache, fever, fatigue, and weight loss. The most common neurological symptoms were motor deficits (34 of 40, 85%), all of whom had ataxia. Other frequently observed symptoms included dysarthria (16 of 40, 40%), behavioral or personality changes (14 of 40, 35%), and cognitive impairment (9 of 40, 22.5%), dizziness (7 of 40, 17.5%), dysgeusia (5 out of 15, 33.3%). Epilepsy developed in four patients (10%), while sleep disturbances and visual disturbances were each reported in two patients (5%). One patient experienced a decreased level of consciousness, and another had sensory impairment. Notably, one child presented with hair and eyebrow loss prior to the diagnosis of anti-mGluR1 encephalitis, which was resolved after immunotherapy. Malignant tumors were identified in nine patients (22.5%), four of whom had Hodgkin lymphoma. The remaining five patients had other malignancies, including mycosis fungoides with prostate adenocarcinoma, testicular seminoma, cutaneous T-cell lymphoma, acute lymphocytic leukemia, and mantle cell non-Hodgkin’s lymphoma. There was no significant difference in tumor association between males and females (OR = 0.750, 95% CI 0.169–3.333, *p* = 0.705). Additionally, 16.7% (*n* = 6/36) of the patients were diagnosed with autoimmune diseases other than anti-mGluR1 encephalitis, such as multiple sclerosis, Hashimoto’s thyroiditis, Sjögren’s syndrome, and pernicious anemia.

**TABLE 1:** Clinical features of 40 autoimmune encephalitis patients with mGluR1 antibodies.

Variable(s)	No. of patients	%
Total	40	100
**Demographics**		
Median age at onset (IQR, years)	50.5	-
Female	20/40	50
Tumors	9/40	22.5
Hodgkin disease	4/40	10
Other tumors	5/40	12.5
Autoimmune disorders	6/40	15%
Prodromal symptoms	10/40	25
Headache	6/40	15
Fever	6/40	15
Fatigue	4/40	10
Weight loss	3/40	7.5
Nausea	3/40	7.5
Flu-like symptoms	1/40	2.5
Shingles	1/40	2.5
Diarrhea	1/40	2.5
**Neurologic symptoms**		
Movement disorders	34/40	85
Ataxia	34/40	85
Tremor	7/40	17.5
Dystonia	4/40	10
Myoclonus	2/40	5
Dysarthria	16/40	40
Behavioral or personality changes	14/40	35
Irritability/mood changes	11/40	27.5
Psychosis/hallucination	5/40	12.5
Cognitive deficits	9/40	22.5
Memory	5/40	12.5
Attention	3/40	7.5
Executive	2/40	5
Spatial orientation	1/40	2.5
Dizziness	7/40	17.5
Visual deficits	6/40	15
Dysgeusia	5/15	33.3
Seizures	4/40	10
Focal seizures	3/40	7.5
Generalized seizures	1/40	2.5
Sleep disturbances	2/40	5
Decreased level of consciousness	1/40	2.5
Sensory disorders	1/40	2.5
**Other symptoms**		
Hair and eyebrow shedding	1/40	2.5
CSF analyses	35/40	87.5
CSF abnormalities	22/35	62.9
CSF pleocytosis	17/35	48.6
CSF OCB (+) or increased IgG index	14/35	40
**mGluR5 antibodies testing results**		
Positive in serum	36/40	90
Positive in CSF	28/40	70
Positive in both CSF and serum (paired samples)	25/40	62.5
Brain MRI at onset or relapse	37/40	92.5
Cerebellar atrophy	18/37	48.6
T2/FLAIR hyperintensities	8/37	21.6
Abnormal EEG	11/22	50
Diffuse slowing	6/11	54.5
Epileptiform discharge	5/11	45.5
**Treatment**		
Immunotherapy	35/40	87.5
First-line immunotherapy	34/35	97.1
IVMP (corticosteroids)	28/35	80
IVIG	24/35	68.6
PE	5/35	14.3
Second-line immunotherapy	17/35	48.6
Rituximab	11/35	31.4
Cyclophosphamide	6/35	17.1
Mycophenolate mofetil	5/35	14.3
Azathioprine	5/35	14.3
Tacrolimus	1/35	2.5
Ofatumumab	1/35	2.9
Hydroxychloroquine	1/35	2.9
Two or more second-line treatments	12/35	34.3
Cancer treatment	9/40	22.5
No immunotherapy treatment	5/40	12.5
**Outcome at the last follow-up**		
Median time from onset to last follow-up (months)	38	-
Partial recovery	20/40	50
Complete recovery	10/40	25
Lack of improvement	8/40	20
Death	2/40	5
Relapse	10/40	25

Detailed CSF analysis was performed in 35 patients (87.5%). Of these, 22 patients (62.9%) demonstrated CSF abnormalities. Seventeen patients (48.6%) exhibited elevated CSF leukocyte count [median 6/L, range 2–214/L]. Oligoclonal bands or elevated IgG index were present in 14 patients (40%). All patients tested positive for mGluR1 antibodies in either CSF or serum samples. The mGluR1 antibodies were found in the serum of 36 patients (90%) and in the CSF of 28 patients (70%). Notably, 25 patients (62.5%) showed concurrent positivity for mGluR1 antibodies in both CSF and serum. EEG abnormalities were observed in eleven cases (50%) among the cohort of 22 participants, comprising three adult patients with epileptiform discharges (all presenting focal seizures) and four patients with diffuse slowing (one child with generalized seizures and three adults without seizures). Neuroimaging data were available for 37 patients (92.5%) through initial and/or follow-up evaluations. Cerebellar atrophy was identified in 18 patients (48.6%), while T2/FLAIR hyperintensities were noted in eight patients (21.6%).

The first-line therapeutic regimen typically involves IVIG, plasma exchange (PE), and IVMP. For refractory cases or suboptimal responders, second-line interventions may encompass alternative immunosuppressive agents (e.g., cyclophosphamide, rituximab) or targeted biologic therapies. Thirty-four patients (85%) received first-line immunotherapy. Second-line immunotherapy was initiated in 17 patients (42.5%), including one patient who received rituximab as primary treatment without prior first-line therapy, yet demonstrated no clinical improvement. Twelve patients (30%) received two or more second-line immunotherapies. All patients with concomitant malignancies received appropriate oncological treatment. Among the five patients (12.5%) who did not undergo immunotherapy, two presented with malignant tumors (one achieving partial recovery and one showing no improvement). Of the remaining three patients without immunotherapy, one died and two failed to achieve clinical recovery. At the final follow-up assessment (median duration: 24 months), the clinical outcomes were as follows: complete recovery in 10 patients (25%), partial recovery in 20 patients (50%), no improvement in 8 patients (20%), and mortality in 2 patients (5%) (Figure 4B). Among patients who received second-line therapy, three patients (17.6%) did not recover. Disease relapse occurred in 10 patients (25%), with a median time to first relapse of 3 months (range 1.5–108 months).

The functional outcomes, as assessed by mRS, demonstrated significant improvement at the last follow-up visit compared to disease peak. The median mRS score decreased from 4 (at disease peak) to 2 (at last follow-up). According to the final mRS assessment, 27 patients (67.5%) had good outcome, and 13 patients (32.5%) had poor outcome (Table 2). There was no statistical difference in mRS scores between the two groups at peak of anti-mGluR1 encephalitis (median 4 vs. 4, *p* = 0.941). Comparative analysis revealed that patients with good outcomes showed significantly higher rates of first-line treatment administration compared to those with poor outcomes (96% vs. 62%, *p* = 0.017). Additionally, the median duration from onset to last follow-up was substantially shorter in the good outcome group (17 months) than in the poor outcome group (36 months) (*p* = 0.038). There were no significant differences between the two groups regarding demographic characteristics, prodromal symptoms, neurological symptoms, combined tumors, CSF analysis, and cerebral MRI.

**TABLE 2:** Comparison of autoimmune encephalitis patients with mGluR1 antibodies according to good or poor outcome.

Variable(s)	Good outcome	Poor outcome	OR	95% CI	P
Patients (total 16), n (female/male)	27 (13/14)	13 (7/6)			
**Demographics**					
Median age at onset (IQR, years)	50	58	0.796	0.211, 2.998	0.736
Female, *n* (%)	13 (48%)	7 (54%)	0.796	0.211, 2.998	0.736
**Comorbidity**					
Tumor, *n* (%)	6 (22%)	3 (23%)	0.952	0.197, 4.611	0.952
Prodromal symptoms, *n* (%)	9 (33%)	3 (23%)	1.667	0.365, 7.607	0.510
**Neurologic symptoms, *n* (%)**					
Dysarthria	11 (41%)	5 (38%)	1.100	0.284, 4.267	0.890
Dysgeusia	3 (11%)	2 (13%)	0.688	0.100, 4.719	0.703
Dizziness	3 (11%)	4 (30%)	0.281	0.052, 1.511	0.139
Behavioral or personality changes	9 (33%)	5 (38%)	0.800	0.202, 3.162	0.750
Cognitive deficits	7 (26%)	3 (23%)	1.167	0.247, 5.502	0.846
Sleep disturbances	0	2 (15%)	0.084	0.004, 1.882	0.118
Seizures	3 (11%)	1 (8%)	1.500	0.141, 15.996	0.737
Decreased level of consciousness	1 (4%)	0	1.528	0.058, 40.092	0.799
Movement disorders	24 (89%)	11 (85%)	1.455	0.212, 9.984	0.703
Sensory disorders	0	1 (8%)	0.152	0.006, 3.985	0.258
**Other symptoms, *n* (%)**					
Hair shedding	1 (4%)	0	1.528	0.058, 40.092	0.799
**CSF analyses, *n* (%)**					
CSF pleocytosis	13 (48%)	4 (31%)	2.089	0.516, 8.464	0.302
CSF OCB (+) or increased IgG index	10 (37%)	4 (31%)	1.324	0.322, 5.439	0.697
**Brain MRI at onset or relapse, *n* (%)**					
T2/FLAIR hyperintensities or meningeal enhancement	7 (26%)	1 (8%)	4.200	0.459, 38.445	0.204
Cerebellar atrophy	10 (37%)	8 (62%)	0.368	0.094, 1.437	0.150
**mGluR5 antibodies results, *n* (%)**					
Positive in CSF	22 (81%)	7 (54%)	3.771	0.876, 16.241	0.075
Positive in serum	24 (89%)	12 (92%)	0.667	0.063, 7.109	0.737
Positive in both CSF and serum (paired samples)	19 (70%)	6 (46%)	2.771	0.706, 10.882	0.144
**Immunotherapy, *n* (%)**					
First-line immunotherapy (IVMP, IVIg, PE, oral prednisone), *n* (%)	26 (96%)	8 (62%)	16.250	1.648, 160.243	**0.017**
Second-line immunotherapy (RTX, MMF, among others), *n* (%)	12 (44%)	5 (38%)	1.280	0.332, 4.942	0.720
Severity (mRS score) at peak of the disease (median)	5	5	0.947	0.225, 3.993	0.941
**Outcome at the last follow-up**					
Median time from onset to last follow-up (IQR, months)	17 (2.5–168)	36 (4–168)	0.222	0.054, 0.923	**0.038**
Last follow-up mRS score (median)	1	4	0.001	0.000, 0.036	**0.001**

Bold values indicate statistical significance (*P* < 0.05).

## 4 Discussion

This study described a novel case of anti-mGluR1 encephalitis that potentially expanded both the clinical phenotype spectrum and therapeutic repertoire for this rare disease. Notably, this represents the first documented instance of anti-mGluR1 encephalitis successfully treated with ofatumumab, demonstrating good clinical outcomes. Compared with rituximab, ofatumumab is a fully human anti-CD20 monoclonal antibody (Sanford and McCormack, 2010). Patients do not need to receive the treatment of prophylactic antihistamines and acetaminophen prior to dosing. In addition, compared with rituximab only binding to one epitope of CD20, ofatumumab binds to different conformational epitopes (Sanford and McCormack, 2010). Furthermore, a comprehensive analysis of 40 reported cases was conducted to characterize the clinical manifestations of anti-mGluR1 encephalitis and identify prognostic factors influencing clinical outcomes. In the summarized case series, there was no significant gender predilection in anti-mGluR1 encephalitis, corroborating previous findings (Christ et al., 2019). The peak incidence occurred between 40–59 years (50% of cases), while pediatric patients (under 18 years) accounted for 15% of the cohort. Malignancies were identified in 22.5% of patients, among which Hodgkin lymphoma constituted one-third of cases. It is not clear whether malignancy is involved in the pathogenesis of anti-mGluR1 encephalitis, but ongoing oncological surveillance is strongly recommended (Spatola et al., 2020). Additionally, 15% of patients presented with concurrent autoimmune disorders, suggesting a potential immunological association.

Prodromal symptoms were observed in only 25% of patients prior to the manifestation of neurological symptoms. Cerebellar ataxia was the most prominent symptom and present in 85% of cases. These results were similar to previous findings (Christ et al., 2019). This may be because mGluR1 is highly expressed in the cerebellum (Ichise et al., 2000). Another study reported that mutations in the GRM1 gene encoding mGluR1 caused progressive cerebellar ataxia (Cesaroni et al., 2024). Beyond ataxia, the summarized cases revealed various movement disorders, including tremor, dystonia, and myoclonus. It was not ignored that 40% patients had dysarthria. In addition, one third patients may have Psychiatric manifestations, ranging from negative symptoms (e.g., apathy, reduced verbal output) and positive symptoms (e.g., irritability, mania, hallucinations). One fifth anti-mGluR1 encephalitis cases had cognitive impairment. The memory deficit was the most frequently affected cognitive domain. A distinctive finding was taste loss in 12.5% of cases, which may be a diagnostic clue. Other common neurological manifestations included dizziness, visual deficits, seizures, sleep disturbances, decreased level of consciousness, and sensory disturbances, aligning with previous research findings (Christ et al., 2019).

Although most patients showed increased CSF leukocytosis or elevated IgG index in CSF analysis, these parameters were normal in 37.1% of patients, which was consistent with previous reports (Chen et al., 2024). This finding underscores the importance of anti-mGluR1 antibody screening in suspected cases. Notably, two patients in the summarized case series presented with elevated IgG indexes and detectable antibodies in the CSF but not in serum (Bien et al., 2020; Vinke et al., 2022), suggesting possible intrathecal antibody synthesis (Chefdeville et al., 2016). Neuroimaging studies demonstrated cerebellar atrophy in nearly half of patients, both at disease onset and during follow-up evaluations. This highlights the necessity for serial brain MRI assessments. EEG abnormalities were detected in 50% of patients. Five patients did not receive immunotherapy. None of them fully recovered. Previous studies have indicated that patients diagnosed with anti-mGluR1 encephalitis have a poorer prognosis compared with other autoimmune encephalitis, such as anti-mGluR5 encephalitis, anti-NMDAR encephalitis, or anti-LGI1 encephalitis (Spatola et al., 2020). This case series analysis revealed complete recovery in only 25% of patients, coupled with a substantial relapse rate of 25%.

Comparative analysis of clinical outcomes revealed distinct prognostic patterns between the good and poor outcome groups. The good outcome group demonstrated significantly higher rates of first-line immunotherapy administration. Additionally, the poor outcome group showed prolonged follow-up duration compared to the good outcome group. These findings suggest that early initiation of first-line immunotherapy may serve as a significant prognostic indicator for clinical outcomes.

The current study has several limitations. This study is retrospective study, which increases the risk of bias. In addition, the lack of standardized reporting in previous case studies may have resulted in incomplete clinical data, as evidenced by missing CSF analysis results in some cases. Furthermore, given the rarity and scarcity of this disease, it is difficult to analyze the effectiveness of treatment and prognostic factors in detail. In the future, prospective studies are needed to provide more effective information for this rare condition.

## 5 Conclusion

Anti-mGluR1 encephalitis is an immune-mediated neurological disorder that requires early diagnosis and timely immunotherapy to optimize clinical outcomes. The disease demonstrates a predilection for middle-aged individuals, with peak incidence occurring between 40–59 years. Complete recovery was attained by merely 25% of the cohort. Administration of first-line immunotherapy appears to be a significant prognostic determinant for good outcomes. Nevertheless, more research data are urgently needed to diagnose and treat this rare anti-mGluR1 encephalitis.

## Data Availability

The original contributions presented in this study are included in this article/supplementary material, further inquiries can be directed to the corresponding authors.
